# Development of the User Requirements for the Canadian WildFireSat Satellite Mission

**DOI:** 10.3390/s20185081

**Published:** 2020-09-07

**Authors:** Joshua M. Johnston, Natasha Jackson, Colin McFayden, Linh Ngo Phong, Brian Lawrence, Didier Davignon, Martin J. Wooster, Helena van Mierlo, Dan K. Thompson, Alan S. Cantin, Daniel Johnston, Lynn M. Johnston, Meghan Sloane, Rebecca Ramos, Tim J. Lynham

**Affiliations:** 1 Canadian Forest Service, Great Lakes Forestry Centre, 1219 Queen St. E., Sault Ste. Marie, ON P6A 2E5, Canada; daniel.thompson@canada.ca (D.K.T.); alan.cantin@canada.ca (A.S.C.); daniel.johnston@canada.ca (D.J.); lynn.johnston@canada.ca (L.M.J.); meghan.sloane@canada.ca (M.S.); rebecca.catherine.ramos@gmail.com (R.R.); tim.lynham@gmail.com (T.J.L.); 2 Canadian Space Agency, 6767 Route de L’aéroport, Longueuil, QC J3Y 8Y9, Canada; natasha.jackson@canada.ca (N.J.); linh.ngophong@canada.ca (L.N.P.); brian.lawrence@canada.ca (B.L.); helena.vanmierlo@canada.ca (H.v.M.); 3 Ontario Ministry of Natural Resources and Forestry, Aviation, Forest Fire and Emergency Services, 95 Ghost Lake Rd, Dryden, ON P8N 2Z5, Canada; Colin.McFayden@ontario.ca; 4 Environment and Climate Change Canada, Canadian Meteorological Centre Operations, 2121 route Transcanadienne, Dorval, QC H9P 1J3, Canada; didier.davignon@canada.ca; 5 Department of Geography, Leverhulme Center for Wildfires, Environment and Society, NERC National Centre for Earth Observation, King’s College London, London WC2B 4BG, UK; martin.wooster@kcl.ac.uk

**Keywords:** wildfire, wildfire management, satellite design, wildfire monitoring, wildfire detection, air quality, carbon emissions, user requirements, wildland fire, forest fire, earth observation, remote sensing

## Abstract

In 2019 the Canadian Space Agency initiated development of a dedicated wildfire monitoring satellite (WildFireSat) mission. The intent of this mission is to support operational wildfire management, smoke and air quality forecasting, and wildfire carbon emissions reporting. In order to deliver the mission objectives, it was necessary to identify the technical and operational challenges which have prevented broad exploitation of Earth Observation (EO) in Canadian wildfire management and to address these challenges in the mission design. In this study we emphasize the first objective by documenting the results of wildfire management end-user engagement activities which were used to identify the key Fire Management Functionalities (FMFs) required for an Earth Observation wildfire monitoring system. These FMFs are then used to define the User Requirements for the Canadian Wildland Fire Monitoring System (CWFMS) which are refined here for the WildFireSat mission. The User Requirements are divided into Observational, Measurement, and Precision requirements and form the foundation for the design of the WildFireSat mission (currently in Phase-A, summer 2020).

## 1. Introduction

Global monitoring of wildfire emissions is supported by the network of geo-stationary weather satellites [1]. Finer resolution polar orbiting systems provide further support by correcting for observational biases [2,3,4], and are more commonplace in direct wildfire management applications. Even still, the use of satellite data in real-time emergency management decision-making remains rare, partially due to the latency of satellite wildfire data [5,6,7]. Furthermore, at high latitudes the geostationary network is challenged by rapidly degrading spatial resolution and atmospheric transmission [1,4], leaving wildfire-prone northern boreal regions with limited wildfire monitoring capacity from polar-orbiting systems and a heavy reliance on monitoring from aircraft.

Efforts to enhance the uptake of satellite data in wildfire management have been pursued through satellite design (e.g., [8,9,10]) and purpose-built information systems (e.g., Global Wildfire Information System [GWIS] [11]); additionally, various commercial proposals have been proposed. However, to date no system has successfully delivered end-to-end operational support to address the specific needs of fire managers. In part this is a result of the broad range of wildfire management practices globally and the resulting variation in specific regional requirements for wildfire monitoring. Inability to accurately define the requirements of end-users presents a broad reaching barrier to operational implementation.

Responses to wildfires vary across Canada [12] and range from a “Full Response” (immediate, aggressive initial/sustained attack), to “Monitored Response” (observation and periodic reassessment; [13]), guided either by zonation, or wildfire specific conditions termed “appropriate response” [14]. In situations with increased wildfire activity, the suppression capacity can be rapidly overwhelmed, resulting in escaped wildfires that may burn very large areas [15,16]. These larger wildfires represent only 3% of the number of wildfires in Canada, yet they account for 97% of the area burned [12,17], and require substantially more resources to manage [18,19].

In many higher risk locations, Canadian wildfire management agencies generally rely on the Initial Attack model where wildfires are detected early and suppressed small. The early detection and suppression of wildfires is critical to successful wildfire control resulting in fewer escaped wildfires, therefore reducing impacts and response costs [20]. Wildfire remote sensing has been recognized for its capacity to detect wildfires (e.g., [21]). However, there is a significant gap between what is required for “early” detection (e.g., identifying small sub-canopy wildfires) for wildfires that require suppression and what can be accomplished reliably with satellite remote sensing [22]. Beyond detection, during periods of escalated wildfire activity it is useful to have current intelligence about all ongoing wildfires. Reliable wildfire intelligence is critical for situational awareness and informed decision-making including prioritization and strategic and tactical wildfire response.

Wildfires that are threatening communities and critical infrastructure are prioritized for suppression action over remote wildfires where there is more opportunity for the natural ecological role of wildfire on the landscape [23]. Therefore, these wildfires more frequently grow larger and are generally managed through modified tactics (e.g., continuous mapping and monitoring; [14]). Although satellites are not typically helpful for early detection [22], there is an emerging requirement for large wildfire and regional intelligence gathering to maintain situational awareness during periods of escalated wildfire activity. This intelligence requirement can be met through the proper application of satellite technology, and is broadly described as wildfire “monitoring” here.

Wildfire monitoring is also an essential component of Canada’s efforts to track and predict smoke dispersion from active wildfires. In recent years, wildfire smoke has been the dominant cause of poor air quality for large portions of Canada [24]. The impacts of smoke on communities can necessitate an evacuation, even without a direct threat from wildfire [25,26,27]. This led to the development of methods to derive emissions from satellite-detected wildfires, and to use the emission estimates in smoke dispersion [28,29] and air quality [30] forecast systems. These automated systems require the provision of timely and reliable wildfire activity data, with an emphasis on wildfire events that produce large long-range transportation of smoke, or wildfire events near communities. With additional development, these systems can evolve to incorporate Fire Radiative Power (FRP; MW) measurements as an additional source for the estimation of wildfire emissions (e.g., [31,32]), as is becoming increasingly common throughout the world (e.g., Global Fire Assimilation System (GFAS); [33].

Under climate change a substantial increase in the frequency and intensity of wildfires is expected [34,35]. In particular, northern regions such as Canada are expected to see an increase of wildfire activity related to increases in conditions conducive to extreme wildfire weather [19,36,37,38,39,40,41]. Consequently, frequency of extreme burning days where wildfires are able to escape is also projected to increase [41,42].

**Figure 1:**
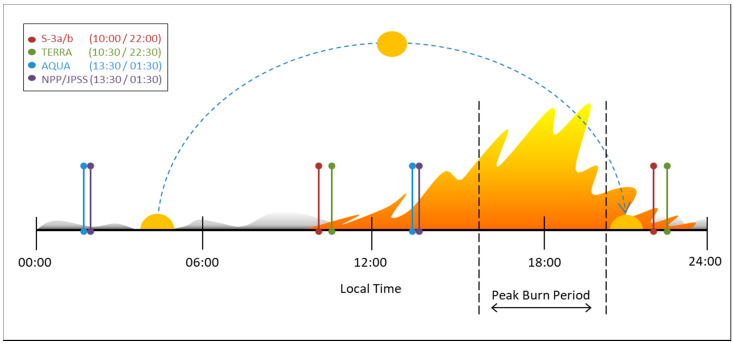
Active-fire satellite overpass time in relation to the diurnal wildfire activity cycle. Wildfire activity varies throughout the day in relation to the changing solar elevation and resulting changes in atmospheric moisture, ambient temperatures, and wind speeds. In general, wildfire activity is dominated by smoldering combustion overnight and in the early morning, and peaks in the late afternoon period centered around 18:00 local time known as the “peak burn period”. Notably, currently available active-fire low-earth-orbiting satellite observations from instruments such as SLSTR (Sentinel-3a/b), MODIS (TERRA/AQUA), and VIIRS (NPP/JPSS) fail to observe wildfires during the most active portions of the day.

In 2019 the Canadian Space Agency (CSA) initiated the development of a dedicated wildfire monitoring satellite “WildFireSat” mission [43]. WildFireSat (WFS) intends to leverage uncooled microbolometer technology developed by the CSA and Institut National d’Optique (INO). An earlier version of this technology called the New InfraRed Sensor Technology (NIRST) was the first mid-wave infrared (IR) microbolometer used in space-based wildfire remote sensing on the 2011 Aquarius SAC-D mission [44]. Following the NIRST experiment, the same technology was used in a feasibility study (referred to as “Phase 0”) to demonstrate the technical feasibility of a cost-effective, dedicated Canadian Wildland Fire Monitoring System (CWFMS) [45]. Since then, the detector technology has continued to evolve (e.g., [46]), while new Low Earth Orbit (LEO) wildfire products (e.g., from the Visible Infrared Imaging Radiometer Suite (VIIRS) and Sea and Land Surface Temperature Radiometer (SLSTR); [47,48]) have filled some temporal coverage gaps (Figure 1), which will improve the feasibility of a targeted wildfire monitoring mission.

Phase-A of the WFS mission is driven by the Mission Requirements [49] which extend from the User Requirements. However, the User Requirements defined in CWFMS [50] required substantial revisions to accommodate the new context of this mission. The aim of this study is to trace the process used to update and re-scope the CWFMS User Requirements for WFS through consideration of emerging science and ongoing end-user consultation (e.g., [51]). This study presents the WFS User Requirements and provides cross reference to their heritage in CWFMS where applicable. We trace the two primary phases of this process: (1) Canadian wildfire management needs are assessed through direct engagement of wildfire management end-users, leading to a set of key Fire Management Functionalities (FMFs); (2) User Requirements for the WFS mission are refined through the integration of the wildfire management needs with the best available scientific techniques. The result of this study is the definition of User Requirements for the first dedicated operational wildfire monitoring satellite, forming the foundation for later stages of mission development.

## 2. Wildfire Management Needs Assessment

Wildfire management agencies generally employ a risk-based approach where the potential impact(s), likelihood, and resulting expected loss or benefit are assessed at the appropriate scale according to the complexity of the wildfire situation [26,52,53]. Decisions often involve multiple decision-makers and stakeholders with varying perspectives concerning risk [23,26]. Decisions are not static and are frequently updated through an iterative process of determining and taking actions, monitoring outcomes, and revising actions until the situation is resolved [53]. When assessing progress in the decision-making cycle, decision-makers require different types of intelligence.

We define wildfire intelligence as information which is collected to support wildfire management activities. This may include current or forecasted information such as: Wildfire behavior, location, size, shape, spatial context (e.g., fuels, topography, proximity to areas of concern, etc.), firefighting resource allocation and use, and wildfire effects and impacts (e.g., social, economic). The type, precision, accuracy, and timeliness of intelligence required varies depending on whether tactical or strategic planning is being conducted.

### 2.1. Wildfire Management Engagement

In the first step of defining the Mission Requirements, Canadian wildfire managers were surveyed to better understand the relative importance of the various wildfire monitoring products and the constraints for their relevance as a source of intelligence in both tactical (e.g., same-day/near-term operations) and strategic (e.g., longer-term preparedness, large wildfire planning) decision-making. Respondents were posed a series of questions regarding potential Earth Observation (EO) data products and asked to consider each in the context of both tactical and strategic decision-making. They were asked to provide the optimal and maximal data latencies (i.e., the time lag from collection to receipt of data when it has most value and the point at which it no longer has value), as well a rating of the importance of each product using a Likert scale (1–5; low–high); respondents were also invited to provide comments to aid in the interpretation of their responses.

#### Survey Results

In total, 55 senior staff from 7 of 13 wildfire management agencies in Canada responded to the survey (Table 1). Most responses were completed individually while a few were coordinated efforts by groups. This sample size is consistent with comparable engagement efforts in similar communities (e.g., [54]). The format of some responses required the data to be reformatted prior to analysis. Group responses were weighted according to the number of people contributing to ensure proportional representation.

Products identified as the most important in both tactical and strategic decision-making were active-fire products; these products are typically derived from thermal observations of active wildfire events, and contribute to assessments of location, spread rate, and intensity (Table 2). Whereas post-fire mapping products (e.g., burned area and severity) were primarily valuable at the strategic level (as well as for non-response management activities, e.g., forest inventory). Tactical intelligence was generally required “as quickly as possible” for all products, with the median of responses indicating 30 min or less (Table 2). For strategic uses the same general preference for active-fire intelligence is found (Table 2); however, slightly longer latencies are acceptable. Data latencies of up to 2 h were indicated as the thresholds for tactical and strategic decision-making. In many cases the information continued to have some value to managers for several hours up to 24 h, but not necessarily for tactical or strategic decision-making. Notably, this consultation process also revealed that the timing of data delivery during daily operations was also a key factor in data utility, due to the cyclical timing of daily decision-making.

### 2.2. Summary of Wildfire Manager Needs

In order to maximize the value of a satellite system for wildfire management, certain features were highlighted through additional comments provided during the end-user consultation. These features included: Fast and consistent data delivery, mapping of active and inactive wildfire areas, smoke and air quality information, wildfire behavior, and threat estimates, as well as detection in remote regions.

#### 2.2.1. Fast and Consistent Data Delivery

Daily wildfire management activities follow planning cycles which depend on the scale of management occurring. For example, an incident command team responsible for planning and carrying out wildfire operations on a large wildfire may have different needs for the frequency and timeliness of information than those planning strategic response at a regional, provincial, or national scale. Generally speaking, in order for intelligence to be incorporated into daily planning activities data must reflect the current situation (i.e., low/short latency), but it is also important to receive the information at a consistent time of day to facilitate routine integration.

#### 2.2.2. Mapping of Active and Inactive Wildfire Areas

Although there is a definite interest in the actively spreading portion, intelligence is required for the entire wildfire. The full perimeter of the burned area as well as the active and previously burned area are valuable in wildfire operations. Previously burned areas may still be smoldering and require prolonged suppression, while unburned “islands” in these areas pose a threat for re-burning. Managers also indicated that they were satisfied with the 375 m spatial resolution of the VIIRS I-band wildfire products [47,55], for general applications (though for high complexity incidents fine resolution airborne mapping may also be necessary).

#### 2.2.3. Wildfire Behavior and Threat Estimates

The proximity and threat to interface zones was identified as critical intelligence in the survey (Table 2). Proximity to these zones is achievable through accurate detection and mapping in conjunction with national interface maps [54].

Wildfire behavior observations were considered to have both tactical and strategic value, particularly in terms of estimating the potential threat of a wildfire. Information relating to the rate and direction of wildfire spread (ROS (m s^−1^) and DIR (deg); [56,57]) as well as the Fire Intensity (FI, (kW m^−1^)), are essential to characterizing the behavior of actively spreading wildfires. Johnston et al. (2017) [58] demonstrated that FI can be estimated directly from IR measurements of FRP. Wildfire behavior is of particular interest during the late afternoon “peak burn” period (Figure 1). This information should ideally be paired with the spatial context (e.g., adjacent fuels, topography, and proximity to areas of concern; [59]) to provide estimates of proximity and threat to these areas.

#### 2.2.4. Detection in Remote Regions

Wildfire management practices in Canada vary dramatically across the landscape, and generally in relation to population distributions (e.g., [9,10]). In vast remote areas, wildfire managers do not typically conduct dedicated detection activities due to the decreased likelihood for negative impacts from wildfire, and the higher cost and operational complexity of these patrols (e.g., [60]). Space-based EO is particularly suitable for gathering intelligence in these situations [22]. The value of EO-derived detection of wildfires identified in the survey (Table 2) was highlighted as particularly valuable in these regions in the comments.

#### 2.2.5. Smoke and Air Quality Information

Although smoke management tools were not identified as critical to tactical decision-making in the survey responses, this information is critical for other emergency management operations. Non-fire management users require smoke-related intelligence for critical operations such as evacuation planning [61,62,63], public health forecasting [64], and aviation visibility [65]. Smoke forecasting using tools such as FireWork [30,66] and BlueSky [29,67] are dependent on wildfire size and location information. Other smoke monitoring applications (e.g., Global Fire Assimilation System (GFAS); [33]) require FRP [31,32] as a primary input.

The delivery of operational smoke and air quality forecast is a highly automated process. As of 2020, Environment and Climate Change Canada will launch a new air quality forecast twice a day (initiated at 00 and 12 UTC), and smoke forecast every 6 h (00, 06, 12, 18 UTC). Each execution is updated with the latest wildfire information available. The scheduling of forecast executions is tied to the availability of new weather and wildfire emission data, and computing resources. Due to this scheduling, the data latency requirements are less stringent than for wildfire management applications, but the requirement to focus on the peak burn overpass period still persists. Additionally, smoke and air quality applications emphasize a strong interest in smaller wildfire detection, an interest in thermodynamic parameters controlling plume rise and height (e.g., [68,69]), and a larger coverage (e.g., North America).

### 2.3. Fire Management Functionalities:

**Figure 2:**
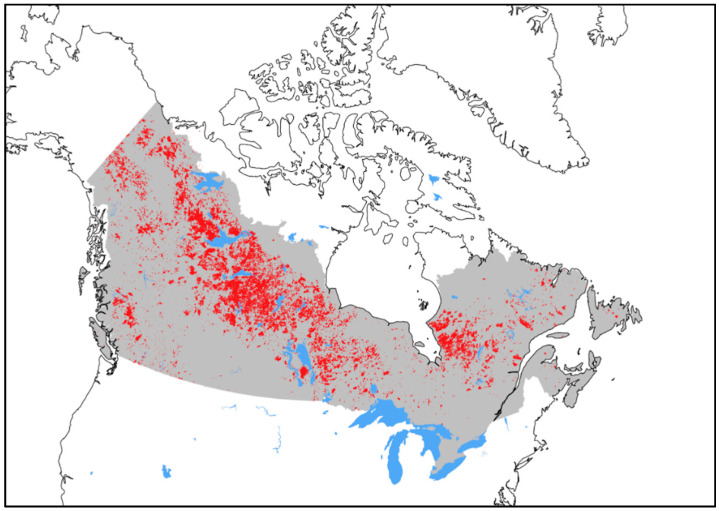
Spatial extent of the WildFireSat Area of Interest (AoI), which includes the entire continuously vegetated extent of Canada (gray). For context, the historic burned area on public, primarily forested lands (1980–2019) is overlaid in red [17]. Although the full AoI is not prone to frequent wildfire activity, the vegetated areas shown here are burnable. The spatial distribution of wildfire within the AoI is expected to change under climate change. The distribution of agricultural burning (primarily confined to the southern half of the AoI) is not shown.

The needs identified above were translated into a set of key Fire Management Functionalities (FMF) necessary to define the User Requirements:

(1) The Area of Interest (AoI) is defined as the whole vegetated Canadian landmass (Figure 2);
(2) Daily (or better) coverage of the AoI at a specific and consistent time of day including peak burn (1600–2000 local time), with data delivery (to end-users) before the start of the operational response period (~0700 local) for overnight observations and before the end of day planning period (~1900 local) for peak burn observations;
(3) Detection and mapping of wildfires and their plumes, specifically:
  i. The ability to detect wildfires with comparable or improved sensitivity to existing satellite systems, and to serve as an early-detection system for remote access wildfires;
  ii. There must be sufficient spatial resolution and geolocation accuracy for locating and mapping wildfires in relation to their previous position and other landscape features;
(4) Estimation of wildfire behavior, specifically:
  i. The ability to collect FRP measurements;
  ii. The ability to characterize sub-pixel wildfire features (e.g., temperature and area);
(5) Compatibility with other available EO data sources and formats;
(6) Near-real-time data, with tactical products to be delivered within 30 min, and a 2-h latency for all end-user products as threshold for utility.

The interconnectivity of the FMFs with the requirements laid out in the User Requirements Document [49] and the Mission Requirements Document [50] is summarized in Table 3. The development and rationale for these requirements is described in the following sections.

## 3. Definition of the User Requirements

The FMFs were considered in order to define EO User Requirements during the CWFMS feasibility study [49] taking into account space system capabilities and limitations, including payload technology capabilities, but without targeting a specific mission scope. With the initiation of the WFS mission, the CWFMS User Requirements were further refined to correspond to a specific mission scope and matching level of funding to produce the Mission Requirements specific for WFS [50]. A simple approach to defining these requirements would be to guarantee success by over prescribing the needs of the mission. However, this is not a programmatically feasible approach as it inflates the mission cost and complexity. In this study, we acknowledge that programmatic constraints will necessitate trade-off analysis during the implementation of the mission. As such, many of the User Requirements are stated as both “goal” (*SHOULD*) and “threshold” (*SHALL*) requirements to reflect optimal and minimum required performances. In this Section the critical WFS User Requirements required to fulfill each of the six FMFs are described in terms of Observation, Measurement, and Precision Requirements for the WFS mission.

### 3.1. Observation Requirements

In this section we describe the critical requirements necessary to observe the target area and report data necessary to achieve the FMFs described in the previous section. Observation requirements described here include those which describe the required coverage and data latency.

#### 3.1.1. Coverage Requirements

Given the success of geostationary wildfire monitoring it could be argued that continuous observations of actively burning wildfires are required in order to rapidly detect new starts. However, persistent observation is typically associated with coarse spatial resolution, which negatively impacts small wildfire detection sensitivity and geographic mapping precision (e.g., [4]). Furthermore, given that FMF-3 does not address detection in high risk areas, this functionality can be met without persistent observation (Table 3). Specifically, one or more satellites with moderate spatial resolution in LEO could accommodate FMF-3.

The ability to observe wildfire behavior (i.e., FI and ROS; FMF-4) is also linked to observational frequency. ROS measurements are a function of spatial and temporal precision and thus the minimum required revisit time can be estimated based on the spatial resolution and the speed of the wildfire being observed [56,71,72]. A ROS-driven coverage requirement was considered in the feasibility study for the CWFMS which proposed a constellation of 9 satellites providing ~500 m spatial resolution with a 2-h revisit period [45]. However, given the overpass times of currently available LEO active-fire observations (Figure 1), a stand-alone constellation is not essential to comply with the ROS driven coverage requirement. A strategically positioned satellite with peak burn overpass (FMF-2), that is compatible with the existing systems (FMF-5) would also meet FMF-4 (Table 3).

Daily coverage during the peak burning period for the entire Canadian landmass (FMF-1) with consistent mapping and wildfire behavior data (FMF-3, 4) delivered prior to the end of the day planning period (~19:00 local time; FMF-2) is also necessary to address the full spectrum of these functionalities. These FMFs also suggest the requirement for consistent overpass times to ensure predictable data delivery times. This implies a preference towards the use of a sun-synchronous polar orbit with a Local Time of Ascending Node or Local Time of Descending Node, i.e., local overpass time of ~18:00 (FMF-2, 6). Notably, the requirement to consistently observe the full AoI (Figure 2) further suggests that the required revisit period cannot be met through instrument pointing or satellite maneuvering as this will cause coverage gaps elsewhere in the AoI.

The User Requirements for coverage are summarized in Table 4. Temporal resolution requirements are specified with the goal of covering the full AoI (Figure 2) on a daily basis. Allowances are made for incidental coverage gaps provided they do not persist for multiple days (Table 4). The temporal resolution is further constrained to ensure that the daily coverage is provided strategically during the peak burn period (Table 4; Figure 1). Additionally, a minimum swath width is defined to ensure that each overpass is capable of observing a complete wildfire area (centrally positioned in the frame), to further support the mapping requirements of FMF-3 (Table 4).

#### 3.1.2. Latency Requirements

The time sensitivity of active-fire data has been consistently highlighted by the end-users and is reflected in FMF-6 (Table 3). The requirement for near-real-time (NRT) data latency is also necessary to ensure that peak burn observations (e.g., ~18:00 local) are delivered to end-users prior to the end of the daily planning period. A threshold requirement for data latency of 30 min is applied to meet these requirements. This latency was selected as a threshold as it was deemed technically achievable; however, the goal latency is to approach real-time if possible. A downlink priority band list (based on minimum requirements for active-fire detection calculations) is provided in the event that not all data can be downlinked in NRT (Table 4).

### 3.2. Measurement Requirements

In this section we define the measurements required from the payload to provide sufficient data to meet the FMFs. Specifically, this section explores instrument band requirements, as well as their associated spatial resolutions and dynamic ranges (Table 5). Information pertaining to band sensitivity and performance is presented in Section 3.3 (Precision Requirements).

In order to meet FMF-3 and -4 the instrument payload must meet all the requirements for active-fire monitoring. This necessitates the ability to detect new wildfires, as well as map their active wildfire areas and behavior, thereby supporting smoke and air quality modeling. This requires the instrument to collect thermal observations to conduct wildfire detection and characterization analysis (e.g., [32,73,74]). It also requires multi-spectral data from a suite of Mid-, and Long-Wave infrared (MWIR and LWIR) combined with Near-infrared (NIR) and Visible (VIS) bands at a minimum (e.g., [47,74,75]). The IR and VIS-NIR bands which observe primarily emitted and reflected energy, respectively, are treated as two complimentary data sets, and are described separately here. 

#### 3.2.1. MWIR and LWIR Band Requirements

The MWIR and LWIR bands are central to the detection of wildfires using their thermal radiance. These bands are also essential in the measurement of FRP (MW), which is a key parameter in wildfire behavior estimation (e.g., [58,76]), and smoke plume emissions (e.g., [31]). FRP calculation can be achieved with single band measurements in the MWIR [32]. However, the LWIR remains essential in the detection of wildfire pixels for FRP analysis as well as in interrogating sub-pixel characteristics of wildfire pixels (e.g., effective wildfire temperature and area; [73]).

Optimal MWIR band placement for wildfire detection and FRP measurement is a narrow window of observation centred at 3.9 µm (e.g., MODIS, Band 21). Wider spectral bands have also been demonstrated to be effective (e.g., SLSTR, Figure 1), provided that they enter into the CO_2_ band at 4.2 µm, and avoid encroaching significantly below 3.5 µm where solar reflection becomes a stronger contributor. These parameters are reflected in the MWIR spectral band requirements in Table 5. The LWIR band’s function of supporting contextual detection analysis and sub-pixel analysis affords more freedom in precise band placement. The LWIR band is specified to ensure that it falls within an atmospheric window in the 8–14 µm range (Table 5), providing sufficient spectral separation for MWIR-LWIR differential analysis.

**Figure 3:**
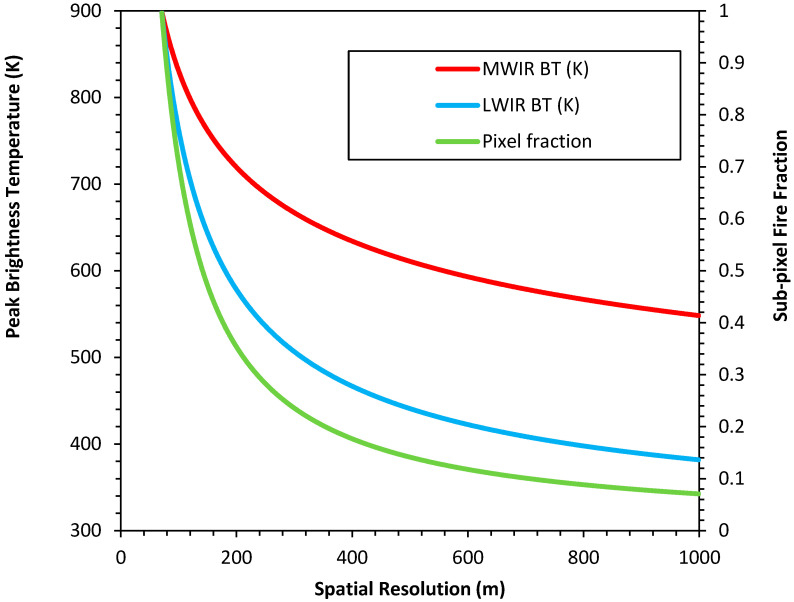
Simulated Infrared (IR) band saturation temperatures as a function of pixel spatial resolution and sub-pixel wildfire area. This simulation assumes a flame front depth of 50 m with mean temperature of 900 K extending diagonally across the pixel. Sub-pixel wildfire area was computed and the saturation temperatures were defined based on the peak pixel brightness temperatures (BT) at a given spatial resolution (Table 5). The saturation temperatures change with spatial resolution in both the LWIR and MWIR, e.g., at 250 m the MWIR and LWIR saturation temperatures are ~690 and ~540 K, respectively, while at 500 m the MWIR and LWIR saturation temperatures are ~610 and ~440 K, respectively. No atmospheric effects on the signal are included in this simulation.

FRP measurement and sub-pixel characterization also requires measurement over the full dynamic range of the scene. Dynamic ranges for the MWIR and LWIR bands (Table 5) were defined in Brightness Temperatures (BT; K) at surface level (i.e., without atmospheric attenuation) as the precise spectral bands are not yet specified. Both bands are required to make ambient (~300 K) surface temperature measurements. Given that this satellite will be designed to observe Canadian wildfires during the peak burn period, the saturation point was defined in anticipation of observing the extreme intensities of Canadian boreal crown wildfires (e.g., [77]) during their peak period. This is a condition not yet achieved through LEO satellites. Therefore, a sub-pixel scene was modeled in which a 50-m deep flame front with net blackbody emission equivalent to ~900 K crosses the pixel area diagonally, while the background area remains ~ 300 K. Under such conditions the saturation point for the MWIR and LWIR spectral bands vary in accordance with their spectral band and with respect to the sub-pixel area of the wildfire. As such, the required dynamic ranges are bound by spectral band selection and spatial resolution (Table 5; Figure 3). Ideally the dynamic ranges for these bands would be stated in spectral radiance ranges at Top of Atmosphere (TOA); however, until the Spectral Response Functions (SRF) and spatial resolutions are known, the modeling exercise in Figure 3 cannot be completed. Atmospheric transmittance modeling as performed for the VIS and NIR bands should be replicated for MWIR and LWIR when appropriate.

Given the interdependency of spatial resolution and dynamic range it is not necessarily advantageous to target unnecessarily fine spatial resolution imagery in the MWIR and LWIR bands. Recent experiences in analysis of the VIIRS active-fire products have demonstrated increasing errors of commission (i.e., false positives) associated with finer scale active-fire detections [55,78]. Johnston (2016) [70] found that ROS measurements could be reasonably estimated under crown wildfire conditions at spatial resolutions as coarse as 500 m. Given FMF-5, MWIR and LWIR spatial resolutions were defined with the intent to be comparable to the VIIRS I-bands (~375 vs. 400 m for WFS) in order to serve as a complimentary data source to the VIIRS data which is captured a few hours earlier in the day (Figure 1).

#### 3.2.2. VIS and NIR Requirements

Although the VIS and NIR bands support contextual wildfire detection (e.g., [74]), their primary contribution is to cloud masking and false positive identification. These applications are less constraining on band selection and dynamic range than the secondary applications of these bands. Although post-fire mapping products are only of strategic value, the ability to map the burned area of a wildfire adjacent to its active portions is an integral part of FMF-3, which requires measurements in the VIS and NIR bands at a minimum (and ideally would include the short-wave IR, see Discussion). VIS and NIR bands are typically used for measures of vegetation “greenness” (e.g., Normalized Difference Vegetation Index; NDVI) to infer vegetation health metrics. Detection of sudden changes in these metrics can be attributed to vegetation disturbance such as wildfires (e.g., [79]). Although numerous systems are capable of mapping global burned area at a relatively fine scale (e.g., [80]), this capability is not typically available as an NRT product in conjunction with active-fire observations as required by FMF-3. Requirements for spectral bands were specified as VIS-red and NIR bands at a minimum. However, the goal band list includes all VIS spectral bands required for forestry observation (Table 5).

Due to the unique overpass time (and consequently unique solar angles) it was not possible to infer dynamic ranges for these bands based off of other burned area mapping systems (which typically have late morning overpasses). To ensure the bands would cover the full dynamic range, a series of MODTRAN simulations were conducted. In these simulations it was assumed that the VIS and NIR bands are expected to be used for imaging surface reflectances during the late afternoon period over the boreal forest. Solar radiation was modeled using a mid-latitude summer atmosphere, with 407 ppmv CO_2_, for a central location in the Canadian landmass (~ latitude 58.0°, longitude 101.0°), at 18:00 local time. Typical boreal forest albedos range from 0.1 to 0.25, while burn scar albedos (~0.05) represent the minimum reflectance of interest, and maximum scene reflectances of ~0.9 are expected from clouds. The threshold VIS and NIR dynamic ranges were defined as those required to observe burned areas up to the peak reflectance of boreal forest, while the goal requirements are defined to include cloud measurements within this range (Table 5).

The objective of burned area mapping with this system is to provide a product suitable for complimenting active-fire detection in FMF-3, not to replace robust techniques used in regional and global carbon accounting (e.g., [81,82,83,84]). With this in mind, fine spatial resolution is not necessary and requirements are relaxed to reduce the volume of data to facilitate NRT calculation. However, as is commonplace in existing systems (e.g., [74,75]), it was deemed desirable to maintain finer scale measurements in the VIS and NIR as compared to the MWIR and LWIR to improve sub-pixel context for cloud masking and resolving cross band misalignments.

### 3.3. Precision Requirements

Precision requirements are defined here to ensure that the measurements provided are of sufficient quality for the analysis required to meet FMF-3 and -4. Table 6 describes instrument band performance requirements, as well as band co-alignment requirements for multi-spectral analysis and geo-location requirements to enable users to locate ground targets.

Although it is clear that the goal is to achieve maximum precision, threshold values for these requirements were defined to support flexibility in design at the mission level as well as overall mission feasibility and affordability. Throughout this analysis the threshold was not intended to represent the optimal design but rather the minimum performance at which the intended analyses and production of the intended data products would be possible (although potentially limited in scope compared with the optimal design).

#### 3.3.1. Sensitivity and Noise Requirements

The goal for all spectral bands is to minimize noise (i.e., maximize signal to noise ratio; SNR), and achieve the highest degree of sensitivity possible (Table 6). However, such requirements have limited value in conducting trade-off analysis and identifying candidate systems and approaches. Given that the primary aim of the mission is to support monitoring activities and only provide detection capacity in remote regions, the sensitivity requirements were not driven by detection requirements. Wildfire managers described satisfaction with the detection capacity of MODIS active-fire products [74] for use in remote regions. Initial screening of candidate pixels in wildfire detection products with comparable sensitivity to MODIS was used to define the thresholds for the MWIR and LWIR bands. The noise and sensitivity requirements in the MWIR also govern FRP accuracy, which was similarly derived with a threshold of achieving the minimum detector performance required to replicate MODIS quality detections. Keeping in mind that the measurement requirements (Table 5) include significantly finer spatial resolution than MODIS, the sensitivity and noise requirements could be relaxed further and still meet the user requirements (Table 6). Additionally, FRP integrity is known to be linked to viewing geometry (e.g., [85]), warranting specification of constraints for off-nadir FRP accuracy (Table 6).

VIS and NIR bands were also defined in terms of their minimum functionality. In this case the threshold criteria were the performance required to adequately generate cloud masks and support contextual wildfire detection calculations. For reference, the threshold SNR values described for the VIS and NIR are comparable to the lower end of what Landsat ETM+ produced [86]. At this performance level the requirement to support active-fire detections will be achieved and would be able to provide data for entry level mapping products.

#### 3.3.2. Co-Registration Requirements

Multi-spectral analysis is essential to the delivery of all of the active- and post-fire products required for the mission. Band-to-band co-registration requirements are defined to minimize band remapping requirements prior to data processing (Table 6), helping to facilitate NRT data delivery under FMF-6. Particularly strong co-registration requirements are imposed on the MWIR and LWIR bands due to their use in bi-spectral sub-pixel analysis [73], which is known to be highly sensitive to band mis-registration errors [87]. Although FRP calculations no longer require the use of the Dozier method [32], the sub-pixel descriptors it produces are required for complete wildfire behavior analysis.

#### 3.3.3. Image Quality and Geo-Location Requirements

A threshold Modulation Transfer Function requirement for the IR optics is defined in Table 6. In order to collect accurate FRP measurements, the MWIR and LWIR bands must collect spatially explicit radiometric measurements. The intent of this requirement is to ensure that the majority of the observed energy originates from within the pixel foot print area.

Wildfire management staff relying on the products from a satellite in an operational setting require that the data is accurately geo-referenced to ensure the location of target wildfires is known with a high degree of certainty (Table 6). Furthermore, FMF-4 and -5 require the data to be compared to observations made from other sources; this cross-platform data fusion necessitates precise geo-location to enable accurate comparisons [88].

## 4. Discussion

The aim of this study was to define requirements for a wildfire monitoring satellite system explicitly to support wildfire management. To do so we surveyed experienced wildfire managers with various specialties in order to better capture their needs in the form of the six FMFs. These functionalities were then used to guide the definition of the WFS User Requirements based on current scientific and technical capabilities.

This study does not describe all factors or analyses considered throughout the process of defining the User Requirements for WFS. Some decisions were made in defining the scope and thus impacted the entire process. For example, the assumption of a LEO satellite mission was taken as the baseline. However, it could be argued that Highly Elliptical Orbiting (HEO) satellite constellations are better suited for the Canadian AoI (e.g., [89,90]), although they are far more costly.

Further, many active- and post-fire products exploit the availability of Short-Wave infrared (SWIR) spectral bands for overnight active wildfire detection and burn severity mapping. Although this spectral band has been considered it was not deemed essential to deliver the FMFs so it was not included as an essential requirement for the mission, though it does remain a goal.

The greatest challenge throughout this process has been defining threshold requirements. It is a simple task to identify the ideal system, payload, and detector. However, determining the limit beyond which a system will no longer be functional is challenging. There is little precedence for marginal systems which can be drawn from. Definition of end-user “usefulness” happens gradually and there is rarely a single threshold of usefulness, particularly when introducing new capabilities. Ultimately, numerous trade-off analyses must be carried out by the Space Team and the User and Science Team where prioritization of the various goal and threshold requirements is necessary for mission development.

The inclusion of trade-off criteria that underpin these requirements is essential as budgetary, technical, and practical limitations will inevitably limit the ability to achieve all of the goal requirements. For example, finer spatial resolution will either reduce the swath and therefore coverage or increase the number of pixels and data volume, challenging the latency requirements. Balancing the consequences of competing requirements is difficult to prescribe a priori. Generally, the trade-off criteria hold coverage of the AoI and IR payload performance as the highest priorities, although overall ability to meet the FMFs is the guiding need. As such, the requirements presented here are a documentation of process at this point in time, but are expected to evolve throughout the mission. The intent is for these requirements to be interpreted in close coordination with the User and Science Team throughout the full mission development.

## 5. Conclusions

In this study we provided an overview of the approach taken to understand Canadian wildfire management EO needs and transcribe User Requirements which can be used to develop a purpose-built wildfire monitoring satellite to meet their needs. The User Requirements presented here originate in the requirements for CWFMS [49], which were refined through consultation of scientific (e.g., [51]) and wildfire management users to produce the User Requirements for the WFS mission.

**Figure 4:**
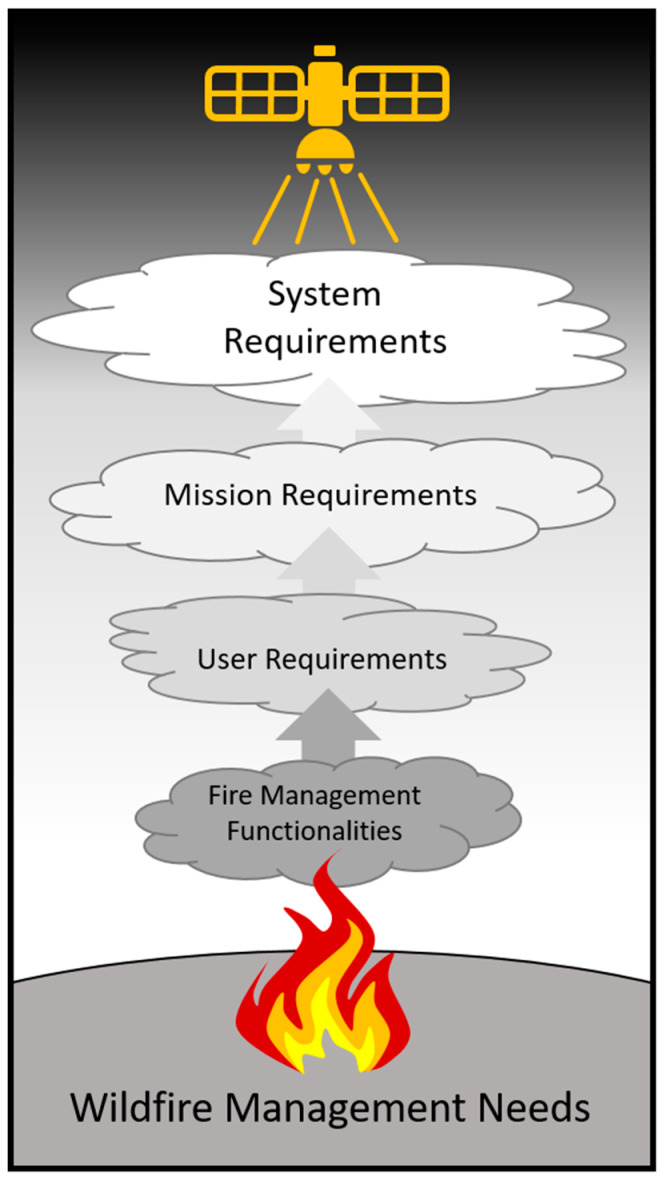
Illustration of the process of defining satellite system requirements based on wildfire management needs in Phase-A of the WildFireSat mission. Fire Management Functionalities (FMFs) are defined qualitatively to capture the Wildfire Management Needs without consideration of space system capabilities or limitations. The FMFs are then translated into User Requirements, which take into consideration space system capabilities required to deliver the necessary data, but do not target any specific mission configuration or scenario. The Mission Requirements define a specific mission scope to address the User Requirements under a specific funding envelope. The Mission Requirements are then used to produce detailed System Requirements describing the technical design of the satellite system. At each level of refinement, the original source of each requirement can be traced back to the end-user’s needs thereby ensuring that the technical design of the satellite system is fundamentally driven by the Wildfire Management Needs.

The translation of the Wildfire Management Needs into User Requirements for WFS is a foundational step in Phase-A of the mission (Figure 4). Through this process we developed qualitative FMFs based on operational wildfire management needs. Considering technical capabilities and limitations allows User Requirements to be defined for a non-specific space system to address the FMFs. When financial and scope considerations are applied to the User Requirements, the WFS Mission Requirements [50] can be specified. Ultimately, Phase-A of WFS culminates by extending the Mission Requirements into detailed technical specifications for the satellite in the System Requirements. As Figure 4 illustrates, although each stage of Phase-A becomes more technically specific, all of the System Requirements are traceable to their origins in wildfire management needs.

WildFireSat aims to deliver a purpose-built operational wildfire monitoring satellite to support wildfire managers as the primary users. To that end, despite the technical and scientific challenges of the mission, the key to operational success remains in the hands of the wildfire management community. In order to achieve meaningful impact in wildfire management operations, the end-user engagement described in this study must continue for the duration of the mission to ensure that wildfire management needs continue to be heard and that wildfire managers develop a sense of ownership in the mission.

## Figures and Tables

**Table 1:** Summary of roles in wildfire management and experience among survey respondents.

Role in Wildfire Management	Description	Percent of Respondents
Aerial Operations	Roles range from Air Operations Branch Director, Air Attack Officers, and Aerial Detection Leaders. In some agencies, these staff coordinate high-level infrared services and other mapping/scanning roles.	17%
Incident Commander (IC) Types 1 and 2	The IC has overall authority and responsibility for conducting incident operations and is responsible for the management of all operations. Levels 1 and 2 are those that lead the most complex wildfire situations.	22%
Incident Commander (IC) Types 3 to 5	Same as above, however the wildfires are less complex.	9%
Plans Section	Roles range from Planning Section Chiefs, Fire Behaviour Analysts, Situation Unit Leaders, GIS, prediction and forecasting services. In some agencies, these staff coordinate airborne infrared services and other mapping operations.	13%
Group Responses	Consisting of agency-selected individuals including skills from roles above.	39%

Table 1: Wildfire management experience levels ranged from 10–39 years with a median of 25 years. Of the 13 Canadian fire management agencies, 7 agencies responded to the survey (specifically: Agencies from Northwest Territories, British Columbia, Alberta, Saskatchewan, Ontario, Québec, and Parks Canada).

**Table 2:** Summary of median latency requirements (minutes) for tactical and strategic decision making. FGM refers Fire Growth Modeling. Ideal and threshold times reflect the median desired data latency and the time at the data was considered no longer valuable for the intended purpose. Likert scoring (1–5; unimportant to very important) is presented as the mode value of the respondent rankings.

Intelligence Type	Product	Tactical Decision Making			Strategic Decision Making		
		Likert Ranking	Ideal Time	Threshold Time	Likert Ranking	Ideal Time	Threshold Time
Active-Fire Observation	Wildfire detection and clustering	5	1	120	5	15	1440
	Rate and direction of spread	4	15	120	4	17.5	300
	Fire intensity	5	15	720	5	12.5	240
Active-Fire Modeling	Smoke mapping	2	30	720	3	45	560
	Suppression effectiveness	3	10	660	3	17.5	660
	Proximity to interface zones	5	5	120	5	15	240
	Risk	3	60	190	3	60	360
	Assimilation into FGM	5	5	720	5	5	720
	FGM prediction	3	20	720	3	17.5	720
	Fuel type verification	3	22.5	720	3	20	720
Pre/Post-Fire Observation	Burned area	3	60	1440	5	60	1440
	Burn severity	2	120	1440	3	90	1440
	Arrival time	4	60	840	3	60	1440

**Table 3:** Primary relationships (not exhaustive) between Fire Management Functionalities (FMFs) and User Requirements from the User Requirements Document [49] (URD). FRP refers to Fire Radiative Power (e.g., [32]).

FMFs	URD Parent References	Rationale
Daily (or better) coverage of the Canadian landmass, with consistent timing and peak burn observation (FMF-1,2)	Temporal resolution (CWFM-URD-0020)	To ensure a minimum of daily coverage is provided
	Peak burn observation (CWFM-URD-0030/40)	To ensure that the daytime overpass falls in the peak burn period
Detection and mapping of wildfires and smoke plumes (FMF-3)	Spatial resolution (CWFM-URD-0080-90)	Constraints are applied to the spatial resolution to ensure adequate ability to resolve active-fire area and to detect change in its position
	Swath (CWFM-URD-0110/120)	Minimum swath width to increase the probability of containing the full wildfire within a single observation
	Spectral bands, dynamic ranges, and sensitivities (CWFM-URD-0150-251)	Minimum spectral bands and performance requirements to conduct cloud masking, wildfire detection and characterization, and burned area mapping
	Band co-registration (CWFM-URD-0260-280)	Wildfire detection, characterization, and mapping requires multispectral measurements. Band co-registration requirements are defined to ensure cross-band analysis is possible across the swath
	Geo-coding (CWFM-URD-0300)	Geographical positioning requirements to enable change detection, comparison to landscape features, and response operations
Measurement of wildfire behaviour (FMF-4)	Spatial resolution (CWFM-URD-0080-90)	Spatial resolution is constrained for optimal ROS measurement [70], and FRP accuracy [71]
	Swath (CWFM-URD-0110/120)	Minimum swath width to increase the probability of containing the full wildfire within a single observation
	Spectral bands, dynamic ranges, and sensitivities (CWFM-URD-0150-251)	Minimum spectral bands and performance requirements to conduct wildfire detection, sub-pixel characterization, and collect FRP measurements
	Band co-registration (CWFM-URD-0260-280)	Sub-pixel wildfire characterization requires multispectral measurements. Band co-registration requirements are defined to ensure cross-band analysis is possible across the swath
Compatibility with other EO systems (FMF-5)	Spatial resolution (CWFM-URD-0080-90)	The spatial resolution range required to meet FMF-3 and FMF-4 is broad, the specific requirements are chosen to closely match VIIRS I-Band spatial resolution
	Peak burn observation (CWFM-URD-0030/40)	A peak burn overpass is required to ensure sufficient temporal offset from the VIIRS overpass time (~ 13:00 local) for optimal Rate of Spread measurement at the specified spatial resolution
Near-real-time data (FMF-6)	Data latency (CWFM-URD-0050)	A data latency of no more than 30 min from overpass to end user delivery is required
	Downlink priority (CWFM-URD-0070)	In the event that not all data can be delivered in near-real-time, priority is given to spectral bands required for active-fire detection and characterization

Table 3: Where URD refers to the User Requirements Document [49], and FRP refers to Fire Radiative Power (e.g., [32]).

**Table 4:** Summary of key Observational (coverage and latency) Requirements as outlined in the User Requirements Document (URD), and refined in the Mission Requirements for WildFireSat. AoI refers to the Area of Interest (Figure 2). See Table 5 for spectral band definitions.

Type	URD Parent Reference	Requirement
Coverage & Temporal Resolution	CWFM-URD-0020	The mission SHALL provide the capability to observe at minimum 97% of all points within the AoI at least once per any 48-h period, and all points within the AoI at least once per 72-h period
		The mission SHALL provide the capability to observe on average 85% of all points within the AoI at least once per any 24-h period
		As a goal, the mission SHOULD provide the capability to observe all points within the AoI at least once per any 24-h period
Peak Burn Observation	CWFM-URD-0030/40	The design SHALL provide observation during each peak burning period
Data Latency	CWFM-URD-0050	Time lag between data acquisition and delivery to user SHALL not exceed 30 min for the MWIR, LWIR, NIR, and VIS (red) for 90% of observations and 24 h for all data
		Time lag between data acquisition and delivery to user SHOULD not exceed 30 min for all data
Downlink Priority	CWFM-URD-0070	Priority downlink SHALL be given to MWIR, LWIR, NIR, and VIS (red) bands
Swath Width	CWFM-URD-0110/0120	Swath width for all spectral bands SHALL be no less than 200 km

**Table 5:** Summary of key Measurement Requirements as outlined in the User Requirements Document (URD), and refined in the Mission Requirements for WildFireSat. TOA refers to Top of Atmosphere based on MODTRAN atmospheric simulations.

Type	URD Parent Reference	Requirement
Spatial Resolution	CWFM-URD-0080	When the spacecraft is nadir-pointing, the design SHALL provide imagery with spatial resolution no larger than: 400 (MWIR, LWIR) and 200 m (VIS, NIR), at the geodetic sub-satellite point 800 (MWIR, LWIR) and 400 m (VIS, NIR), for all pixels
	CWFM-URD-0090	When the spacecraft is nadir-pointing, the design SHOULD provide imagery with spatial resolution no larger than: 300 (MWIR, LWIR) and 150 m (VIS, NIR), at the geodetic sub-satellite point
Spectral Bands	CWFM-URD-0150	The payload SHALL provide at least one band in each of the following channels: MWIR: (3.4–4.2 μm), including include 3.9 μm LWIR: (8.0–9.5 μm) OR (10.4–12.3 μm; preferred) NIR: (0.8–0.9 μm) VIS: (0.6–0.7 μm; red)
	CWFM-URD-0151	The payload SHOULD provide at least one band in each of the following channels: MWIR: (3.5–4.2 μm), including 3.9 μm LWIR: (10.4–12.3 μm) NIR: (0.8–0.9 μm) VIS: (0.6–0.7 μm; red), (0.5–0.6 μm; green), AND (0.4–0.5 μm; blue)
IR Dynamic Ranges	CWFM-URD-0180	Brightness temperatures retrieved in the MWIR band(s) SHALL be in the range of at least 300 to 720 K at 200 m spatial resolution, or 300 to 635 K at 400 m, at surface level
	CWFM-URD-0190	Brightness temperatures retrieved in the LWIR band(s) SHALL be in the range of at least 300 to 580 K at 200 m spatial resolution, or 300 to 470 K at 400 m, at surface level
VIS-NIR Dynamic Ranges	CWFM-URD-0200	The NIR and VIS TOA radiance range SHALL be a minimum of: NIR: 4.0–46.4 (W m^−2^ sr^−1^ μm^−1^) VIS (red): 4.1–25.2 (W m^−2^ sr^−1^ μm^−1^) VIS (green): 2.5–25.8 (W m^−2^ sr^−1^ μm^−1^) VIS (blue): 0.7–16.9 (W m^−2^ sr^−1^ μm^−1^)
		The NIR and VIS TOA radiance range SHALL be a minimum of: NIR: 4.0–139.0 (W m^−2^ sr^−1^ μm^−1^) VIS (red): 4.1–75.5 (W m^−2^ sr^−1^ μm^−1^) VIS (green): 2.5–77.3 (W m^−2^ sr^−1^ μm^−1^) VIS (blue): 0.7–50.5 (W m^−2^ sr^−1^ μm^−1^)

**Table 6:** Summary of key Precision Requirements as outlined in the User Requirements Document (URD), and refined in the Mission Requirements for WildFireSat. SNR refers to the Signal to Noise Ratio, NESR refers to the Noise Equivalent Spectral Radiance, and TOA refers to Top of Atmosphere.

Type	URD Parent Reference	Requirement
Noise	CWFM-URD-0207	The VIS/NIR noise SHALL be: SNR > 30 over the full dynamic range (Table 5; CFWM-URD-0200) for the VIS bands SNR > 40 over the full dynamic range (Table 5; CFWM-URD-0200) for the NIR band
	CWFM-URD-0208	The LWIR band SHALL have NESR < 0.12 (W m^−2^ sr^−1^ μm^−1^) when computed for a TOA scene at 300 K
	CWFM-URD-0209	The MWIR band SHALL have NESR < 0.22 (W m^−2^ sr^−1^ μm^−1^) when computed for a TOA scene at 300 K
	CWFM-URD-0210	The band noise SHOULD be: SNR > 90 over the full dynamic range (Table 5; CFWM-URD-0200) for the VIS bands SNR > 110 over the full dynamic range (Table 5; CFWM-URD-0200) for the NIR bands LWIR and MWIR NESR should be minimized
Sensitivity	CWFM-URD-0220/30	The design SHALL be capable of measuring the background temperature in the MWIR and LWIR channels with a 2σ uncertainty of no more than 5% at TOA for the sub-satellite pixel
		The design SHOULD be capable of measuring the background temperature in the MWIR and LWIR channels with a 2σ uncertainty of no more than 3% at TOA for the sub-satellite pixel
	CWFM-URD-0240	Minimum FRP detectable SHOULD be 5 MW at nadir and 40 MW at edge of swath (up to 45 degree off-nadir)
	CWFM-URD-0250	The uncertainty of the FRP of the center pixel when nadir-pointing SHALL be less than 15% of the FRP, or less than 5 MW (whichever value is larger).
	CWFM-URD-0251	The uncertainty of the FRP of pixels at edge of swath (45 degree off-nadir) when nadir-pointing SHOULD be less than 15% of the FRP, or less than 5 MW (whichever value is larger).
Co-registration	CWFM-URD-0260	Co-registration between MWIR and LWIR bands SHALL be provided within 1/3 MWIR pixel accuracy for all pixels in each of the bands
	CWFM-URD-0270/71	Co-registration between LWIR band and VIS/NIR bands SHALL be provided within 1 LWIR pixel accuracy for all pixels in each of the bands, and SHOULD be within 0.5 LWIR pixel accuracy
	CWFM-URD-0272/73	Co-registration between MWIR band and VIS/NIR bands SHALL be provided within 1 MWIR pixel accuracy for all pixels in each of the bands, and SHOULD be within 0.5 MWIR pixel accuracy
	CWFM-URD-0280	Co-registration between NIR and VIS bands SHOULD be provided within 1-pixel accuracy of either the VIS or NIR band (whichever has the smallest spatial resolution at nadir), for all pixels in each of the bands
Image quality	CWFM-URD-0290	The Modulation Transfer Function for all bands SHOULD be >0.3 for all frequencies below the Nyquist frequency
Geo-location	CWFM-URD-300	Data provided to the users SHALL be tagged with geo-referencing information accurate to within 0.5-pixel resolution
